# Whole genome sequence of four *Streptomyces asiaticus* strains: DSM 41763, DSM 41765, DSM 41767, and DSM 41768

**DOI:** 10.1128/mra.00127-26

**Published:** 2026-04-30

**Authors:** Mohamed E. Hamid, Yvonne Mast, Imen Nouioui

**Affiliations:** 1 Department Bioresources for Bioeconomy and Health Research, Leibniz Institute DSMZ-German Collection of Microorganisms and Cell Cultures 28351, Braunschweig, Germany; 2 Department of Clinical Microbiology and Parasitology, College of Medicine, King Khalid University 48144, Abha, Saudi Arabia; 3 Technische Universität Braunschweig, Institut für Mikrobiologie 26527, Braunschweig, Germany; University of Southern California, Los Angeles, California, USA

**Keywords:** actinomycetes, natural product, bioprospecting

## Abstract

This study presents the genomic features and biosynthetic potential of four *Streptomyces asiaticus* strains—DSM 41763, DSM 41765, DSM 41767, and DSM 41768—and provides insight into the intraspecies diversity. The strains had genome sizes ranging from 11.5 to 11.9 Mbp, with a consistent GC content of approximately 71%.

## ANNOUNCEMENT

Streptomycetes are gram-positive, filamentous bacteria known for their ability to produce a wide range of natural products (1). However, little is known about the intraspecies diversity and their biosynthetic potential (2). In this context, a screening campaign of the DSMZ open culture collection was conducted to identify *Streptomyces* strains that have not yet been assigned to any species, aiming to clarify their taxonomic status and expand the genetic diversity at the species level. Consequently, four *Streptomyces asiaticus* strains—DSM 41763, DSM 41765, DSM 41767, and DSM 41768—were selected as the focus of this study. These strains were originally isolated by Dr. Langkah Sembiring in 1997 from the ectorhizosphere of *Paraserianthes falcataria* in Yogyakarta, Indonesia, and deposited in the DSMZ collection by Prof. Dr. Michael Goodfellow (Newcastle University, UK) in 2000 as *Streptomyces* sp. Detailed information on their history, optimal growth conditions, and morphology can be found in the DSMZ online catalog (https://www.dsmz.de/collection/catalogue/microorganisms/catalogue). The identity and authenticity of the strains were confirmed through near-complete 16S rRNA gene sequence analysis (>1,400 bp). Biomasses, prepared under the growth condition listed in Table 1, were subjected to genomic DNA extraction using a Genomic-tip 100 G−1 kit (Qiagen), following the manufacturer’s instructions. The 16S rRNA gene of the strains was amplified using PCR (3) and sequenced using an Applied Biosystems 24-capillary system, as described in Risdian et al. (4).

**TABLE 1:** Comparative genomic characteristics of *Streptomyces asiaticus* strains

Attribute	*Streptomyces asiaticus* DSM 41763	*Streptomyces asiaticus* DSM 41765	*Streptomyces* asiaticus DSM 41767	*Streptomyces asiaticus* DSM 41768
Growth conditions	DSMZ 65 at 28°C, 6 d	DSMZ 65 28°C, 6 d	DSMZ 84 at 28°C, 6 d	DSMZ 65 at 28°C, 6 d
Genome size	11,868,304	11,751,120	11,538,313	11,696,812
GC content	71	71	71.1	71.1
N50	227,560	444,957	190,713	431,216
L50	14	9	19	10
Number of contigs	298	220	296	200
Number of coding sequences	10,580	10,315	7,628	10,368
Number of RNAs	75	74	71	73
Number of trimmed reads	1,633,096	2,198,582	1,312,782	2,232,104
Genome coverage	63×	86×	51×	89×
Completeness (%)	99.34	99.34	99.34	99.34
Contamination (%)	2.05	1.20	1.14	1.33
GenBank accession numbers	JBPQHC000000000	JBPQGY000000000	JBPQGZ000000000	JBPQHA000000000

Whole genome sequencing of the strains was performed by the MicrobesNG service in Birmingham, UK. The cells were incubated for 25 min at 37°C in the presence of a lysate solution (TE buffer, lysozyme [MPBio, USA], metapolyzyme [Sigma-Aldrich, Missouri, USA], and RNase A [ITW Reagents, Spain]). The cell lysates were then incubated for 5 min at 65°C in the presence of proteinase K (VWR Chemicals, Ohio, USA) and SDS (Sigma-Aldrich) at final concentrations of 0.1 mg/mL and 0.5% vol/vol, respectively. An equal volume of SPRI beads was then added to purify the genomic DNA, which was subsequently resuspended in EB buffer (10 mM Tris-HCl, pH 8.0). The Nextera XT Library Prep Kit (Illumina, San Diego, USA) was used for library construction, following the manufacturer’s instructions with some modifications: the DNA amount and PCR elongation time were increased twofold and to 45 s, respectively. A Hamilton Microlab STAR automated liquid handling system (Hamilton Bonaduz AG, Switzerland) was used for library preparation. Sequencing was performed using an Illumina NovaSeq 6000 (Illumina) (2 × 250 bp kits). The reads were trimmed using Trimmomatic (v0.3) (5) with a sliding window quality cutoff of Q15. *De novo* assembly was performed using SPAdes (v3.7) (6). CheckM analysis (v1.2.4) (7) was used for assessing the quality of the genome (completeness and contamination). The genomes were submitted to GenBank and automatically annotated via PGAP (8–10). Default parameters were used for all software listed above.

The genome sizes of the strains ranged from approximately 11.5 to 11.9 Mbp, with a GC content of ~71%. Further details on the genomic features of the strains are listed in Table 1.

## Data Availability

This Whole Genome Shotgun project of strains DSM 41763, DSM 41765, DSM 41767, and DSM 41768, has been deposited at DDBJ/ENA/GenBank under accession numbers JBPQHC000000000, JBPQGY000000000, JBPQGZ000000000, and JBPQHA000000000, respectively. The genomic data described for strains DSM 41765, DSM 41767, DSM 41768, and DSM 41763 can be freely and openly accessed on GenBank of NCBI under BioProject numbers PRJNA1289108, PRJNA1289111, PRJNA1289114, and PRJNA1289042, respectively. The 16S rRNA gene sequences of strains DSM 41763, DSM 41765, DSM 41767, and DSM 41768 are available in the NCBI GenBank under accession numbers PV960948, PV960949, PV960950, and PV960951, respectively. The raw sequence reads of strains DSM 41763, DSM 41765, DSM 41767, and DSM 41768 have been submitted to the Sequence Read Archive in NCBI (SRX31383377, SRX31383378, SRX31383379, and SRX31383542 respectively).
